# Spatiotemporal cell type deconvolution leveraging tissue structure

**DOI:** 10.21203/rs.3.rs-9589852/v1

**Published:** 2026-06-02

**Authors:** Macrina Lobo, Ziqi Zhang, Xiuwei Zhang

**Affiliations:** 1 School of Computational Science and Engineering, Georgia Institute of Technology, Atlanta, 30332, Georgia, USA.

**Keywords:** deconvolution, 3D, spatial transcriptomics

## Abstract

Spot-based spatial transcriptomics (ST) captures aggregated transcriptomic profiles at spatial locations (spots) in tissues. Deconvolution methods are needed to estimate the proportion of each cell type in every spot, and usually leverage a single cell transcriptomic reference (scRNA-seq). Though there are an increasing number of experiments that profile multiple adjacent tissue slices, no deconvolution method leverages 3D tissue structure. Some methods utilize the 2D spatial organization assuming neighboring spots are similar, which is not the case in heterogeneous environments. Moreover, most methods aggregate reference scRNA-seq profiles of the same cell type, missing subtle cell state variations.

We present SpaDecoder, a parallelized per-spot deconvolution method for multiple neighboring spatial or temporal ST slices that predicts cell type proportions with a matrix factorization-based objective. SpaDecoder uses slice alignment, per-spot spatio-transcriptomic neighborhood inference, and 3D spatial Gaussian kernel weights to effectively leverage 3D structure and adapt to heterogeneous tissue environments. We model individual scRNA-seq profiles, instead of cell type aggregated, to capture cell state variability.

The mathematical framework of SpaDecoder supports several downstream analyses. It uncovers key cell type regions and changing composition across slices, identifies colocalized cell types, imputes spatial gene expression, and predicts 3D spatio-temporal scRNA-seq cell locations. SpaDecoder outperforms other methods on various metrics, datasets, scenarios, and ablations, and yields interpretable biology, showing it harnesses 3D structure and single cell reference profiles to improve deconvolution. SpaDecoder is available at https://github.com/ZhangLabGT/spadecoder.

## Introduction

Higher order multicellular organisms comprise trillions of single cells that are spatially organized into complex tissues with diverse structures performing specific functions. Each cell contains millions of RNA molecules which, when measured via single cell RNA sequencing (scRNA-seq) and mapped to genes, provide insights into the gene expression driving cellular state and function, cell type markers, and identification of novel cell types not evident from tissue morphology. Capturing RNA molecules along with their spatial locations further elucidates the 3D organization of cells into tissues, the spatial expression pattern of genes, as well as intracellular and intercellular communication signaling pathways [1]. These insights are critical to several biological questions such as the underlying mechanisms driving development and healthy cellular processes as well as disruptions due to diseases such as cancer.

Several spatial transcriptomic (ST) technologies have been developed to measure RNA levels along with spatial location at spot, cellular, or sub-cellular resolution by capturing individual RNA molecules [2]. ST technologies typically offer trade-offs between cost, number of genes profiled, spot resolution and the detection efficiency of genes. A single spot may contain 10–200 cells for older ST technologies or 1–30 cells for newer technologies such as Visium from 10x Genomics, or may be at subcellular resolution [3, 4]. Compared to single cell or subcellular spatial omics, multi-cell spots typically capture more genes, at higher detection efficiency and lower cost and is also most abundantly available. However, to utilize this, computational techniques called deconvolution methods, are needed to decode spots into the individual cell types.

Several spatial deconvolution methods have been developed and benchmarked [5–20]. Current approaches utilize scRNA-seq reference atlases [20–23] which provide single cell resolution transcriptome wide profiles of the same tissue region or are reference-free [24–26]. Reference-based methods with appropriately chosen reference datasets typically outperform reference-free methods [9], so it is advantageous to utilize abundantly available scRNA-seq atlases while handling the additional complexity arising out of batch effects between scRNA-seq and spatial datasets due to different sequencing depth, gene detection efficiency, and dropout. The reference should capture a similar section of tissue at the same developmental, healthy or diseased state to prove most effective.

Existing methods have several limitations. First, although an increasing number of experiments profile multiple spatially or temporally proximal tissue slices to study 3D cellular and molecular behavior within their spatial context, no method takes advantage of the available 3D tissue structure for deconvolution. While several methods exist to align multiple 2D slices [27–33], current methods leveraging 3D structure have been confined to a few other problems like spatial domain detection [34], with no documented application in deconvolution. Second, while recent work [9, 35] has leveraged the 2D spatial proximity of spots, under the hypothesis that spatially adjacent spots have similar cell composition, this assumption while true in more homogeneous cellular environments, can face limitations with more complex tissue structures [36]. It is therefore useful to share information across tissue locations that are spatially as well as transcriptionally similar while adapting to region heterogeneity surrounding each spot. Third, among scRNA-seq reference-based methods, most approaches aggregate single-cell profiles across cells of the same type thereby failing to fully utilize the diversity in cell state that arises even among cells from a predefined cell type. Incorporating reference scRNA-seq at single cell resolution would help in this regard.

In order to address the aforementioned challenges, we present SpaDecoder, a deconvolution method for multiple proximal spatial or temporal ST tissue slices, which predicts cell type proportions with parallelized per-spot optimization of a matrix factorization based loss function. It uses 3D spatial Gaussian kernel weights, enabling information sharing within and across slices. The kernel is adaptive allowing the selection of transcriptionally similar neighboring spots, thus avoiding errors due to highly dissimilar neighboring spots. We develop a *local spatially weighted transcriptomic autocorrelation* metric (LSTA) for each spot and perform permutation testing to intelligently select the neighborhood to pool transcriptomic and spatial information, adapting to homogeneous and heterogeneous tissue environments and improving deconvolution. We leverage individual scRNA-seq reference profiles, instead of cell type aggregated, along with their cell type annotations, to account for variability among cells of the same type.

We first establish the deconvolution performance of SpaDecoder qualitatively as well as quantitatively under various scenarios using three spatial datasets [37–39]. SpaDecoder consistently outperforms baseline methods in all scenarios, showing the advantage of using a 3D adaptive kernel and incorporating scRNA-seq reference data at single cell level. Moreover, the learned variables in the matrix factorization framework are interpretable and allow for a range of downstream tasks. To leverage them, we define several metrics and perform downstream analyses to distill SpaDecoder cell type proportions and the learned mapping between scRNA-seq and spatial locations into meaningful biological findings. We showcase the application of SpaDecoder on several downstream tasks. We capture antero-posterior cell type variability across 12 samples or slices of the mouse hypothalamic preoptic region [37], denoise spatial gene expression and predict the expression of unmeasured genes. In human breast cancer tissue [40], we identify colocalized cell types and subtypes in 3D spatial tissue neighborhoods and isolate key tissue regions associated with the corresponding cell types. Finally, we demonstrate the performance of SpaDecoder on a temporal dataset capturing 16–19 post conception weeks (pcw) of human embryonic thymic development [41], where, in addition to identifying cell type regions and colocalized cell types, we predict the 3D spatio-temporal locations of scRNA-seq reference cells.

## Results

### Overview of SpaDecoder

**Fig. 1:**
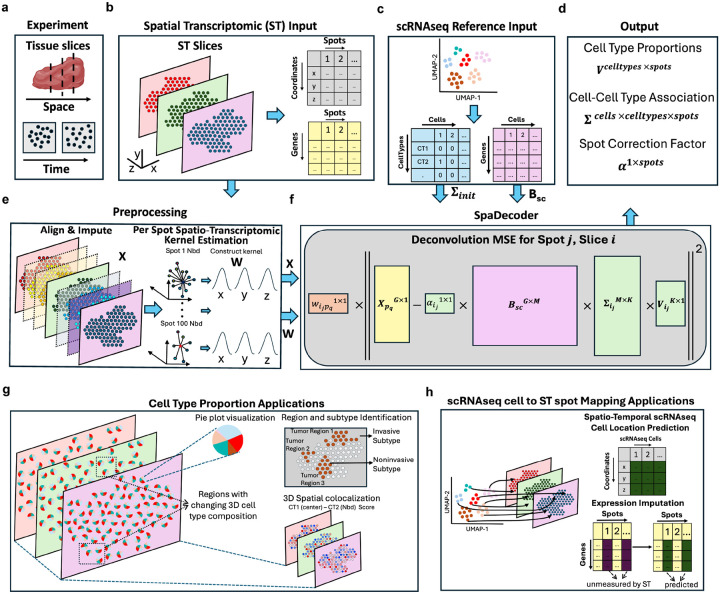
SpaDecoder performs spatio-temporal deconvolution of 3D tissue slices Schematic of the SpaDecoder approach (a) It handles experimental data from multiple tissue slices profiled spatially or temporally. (b) The spatial transcriptomic input “query” comprises a spot by (x,y,z) coordinate matrix and a spot by gene expression matrix. (c) The scRNA-seq reference dataset is used to obtain a cell by gene expression matrix. scRNA-seq cell type annotations are used to obtain an indicator cell by cell type matrix with 1 indicating that the cell belongs to the corresponding cell type and 0 otherwise. (d) For each spot, SpaDecoder outputs cell type proportions which sum to 1, a cell-to-cell-type association matrix whose columns sum to 1, and a spot correction scalar. (e) SpaDecoder preprocessing comprises alignment of multiple slices with moscot [33], imputation of additional intermediate slices, per spot 3D neighborhood selection and spatio-transcriptomic kernel estimation. (f) SpaDecoder objective function for spot j in slice i.G,M and K denote the number of genes, single cells and cell types respectively. $i_{j}$ and $p_{q}$ denote spot j in slice i and spot q in slice p respectively. $w_{i_{j}p_{q}},X_{p_{q}},\alpha_{i_{j}},B_{sc},{𝚺}_{i_{j}},V_{i_{j}}$ denote the 3D spatial Gaussian kernel weights between $i_{j}$ and $p_{q}$, gene expression vector of $p_{q}$, spot correction scalar for $i_{j}$, scRNA-seq gene-by-cell expression matrix, cell-to-cell-type association matrix for $i_{j}$, and cell type proportions in $i_{j}.\;\alpha_{i_{j}},\;{𝚺}_{i_{j}}$, and $V_{i_{j}}$ are learned by optimization. (g) SpaDecoder cell type proportions for each spot are used in spot-level pie chart visualizations, identifying regions with changing cell type composition across slices, pinpointing the spatial extent of each cell type containing region, identifying global and local 3D spatial cell type colocalization. (h) SpaDecoder infers an scRNA-seq cell to spot mapping matrix which is used to predict the spatio-temporal coordinates of single cells, to denoise existing spatial gene expression and predict expression of unmeasured genes.

SpaDecoder is developed for experiments profiling a 3D stack of multiple spatial or temporal spot-based spatial transcriptomic (ST) slices in proximity to each other (Fig. 1a). The input comprises “query” ST spot resolution gene expression and coordinate matrices (Fig. 1b) with a “reference” cell type annotated single cell RNA-sequencing (scRNA-seq) gene-by-cell expression matrix ${(B}_{sc})$ (Fig. 1c). SpaDecoder learns the deconvolution vector $\left(V_{i_{j}}\right)$ containing proportions for each annotated scRNA-seq reference cell type at each spatial spot j in every slice i. SpaDecoder additionally learns a reference cell-to-cell-type association matrix $\left({𝚺}_{i_{j}}\right)$, that represents scRNA-seq cells lying on a continuum of cell states; and a spot correction scalar $\alpha_{i_{j}}$, representing spot-specific batch effects (Fig. 1d). Pre-processing comprises slice alignment to leverage 3D spatial tissue structure; intermediate slice imputation to bridge the gap between slices and augment our dataset; adaptive neighborhood inference to account for homogeneous and heterogeneous tissue regions; and spot-specific 3D Gaussian kernelization of neighborhood spatial distances (Fig. 1e, Methods).

SpaDecoder factorizes the gene expression $X_{i_{j}}$ of spot j in slice i with $B_{sc}{𝚺}_{i_{j}}V_{i_{j}}$ (Fig. 1f). ${𝚺}_{i_{j}}$ encodes cell types in the scRNA-seq dataset and is initialized with cell type annotation indicator variables. To capture subtle cell-state variability among cells of the same type, ${𝚺}_{i_{j}}$ is relaxed onto the simplex representing soft cell-to-cell-type assignment which is learned, along with $V_{i_{j}}$ and $\alpha_{i_{j}}$, through optimization. $B_{sc}{𝚺}_{i_{j}}$ aggregates expression from cells of each type, yielding “pseudo-bulk” cell type profiles which are combined with the cell type proportions in $V_{i_{j}}$ to yield the expression of spot j. The SpaDecoder loss minimizes the weighted distance between the factorized gene expression of the current spot j in slice i and the expression of neighboring spots q in slice p in the 3D tissue space (Fig. 1f, Methods).

Previous work [9, 35] incorporates the 2D spatial neighborhood by assuming spatially neighboring spots are similar and defining a fixed neighborhood for each spot, an assumption that is violated in heterogeneous tissue regions. Instead, we adaptively infer the 3D neighborhood $nbd_{i_{j}}$ for each spot j in slice i (spot $i_{j}$). We obtain a 3D spatial Gaussian kernel $w_{i_{j}p_{q}}$ from within slice spatial and across slice alignment distances between spots $i_{j}$ and $p_{q}$. For each spot $i_{j}$, to identify transcriptionally similar spatially proximal spots q in slice p (spot $p_{q}$), we define a *local spatially weighted transcriptomic autocorrelation* metric (LSTA) consisting of the neighborhood aggregated transcriptomic distances weighted by their corresponding spatial kernel weights $w_{i_{j}p_{q}}$. We perform parallelized permutation testing with LSTA across different transcriptomic neighborhoods, to identify the largest significantly correlated 3D neighborhood $nbd_{i_{j}}$. The selected neighborhood thus adapts to homogeneous and heterogeneous tissue regions. Spots in the selected neighborhood are weighted by the 3D spatial Gaussian kernel $w_{i_{j}p_{q}}$ in the objective function to model decaying similarity with distance (Fig. 1e–f, Methods).

We leverage SpaDecoder inferred cell type proportions (Fig. 1g) and scRNA-seq to ST mapping (Fig. 1h) to solve several downstream tasks addressing different biological questions. From the cell type proportions for each spot, we define two metrics, a global (G-3DSCI) and local (L-3DSCI) 3D Cell Type Spatial Colocalization Index to reveal cell type colocalization patterns. We identify tissue regions with significantly varying cell type composition across slices with permutation testing and the Jenson-Shannon distance metric, and perform connected component analysis to capture distinct spatial regions corresponding to key cell types (Fig. 1g, Methods). We leverage SpaDecoder's reference scRNA-seq cell-to-spatial-spots mapping to annotate fine grained cell types in space, predict the 3D spatio-temporal location of scRNA-seq cells, denoise the expression of measured spatial genes, and predict the expression of unmeasured genes (Fig. 1h, Methods).

### SpaDecoder improves cell type deconvolution on three different mouse tissues

To evaluate the performance of SpaDecoder in various scenarios, we obtain single cell resolution spatial transcriptomic data from three different mouse tissues which we aggregate into square spots of fixed size determined by a parameter and comprising varying number of cells in each spot (Methods). We use spatial cell type annotations from the original publications as ground truth for evaluation. To obtain a 3D stack of tissue slices, for a given sample, we simulate a stack of 10 slices, using a parameter $N_{\text{swap}}$ with higher values indicating greater dissimilarity across 3D slices (Methods). We compare SpaDecoder against top-performing deconvolution methods as identified by benchmarks - CARD [9], Tangram and the single cell reference resolution version of Tangram (Tangramsc) [17] and Cell2location [11] using three established metrics [20]: root mean square error (RMSE), Pearson correlation, and Jenson-Shannon divergence (JSD). The datasets are as follows: (a) Moffitt2018 [37] is profiled using MERFISH on the mouse hypothalamic preoptic region comprising 132 slices (12 samples with a 10-slice simulated stack added on for each) (b) Choi2023 [38] comprises MERFISH on the mouse retina comprising 275 slices (25 original samples) [38] and (c) Haviv2025 [39] comprising 11 Xenium slices (1 original sample) on a mouse model of Leptomeningeal melanoma metastasis. We obtain reference scRNA-seq datasets of the corresponding tissue regions from Moffitt *et al* [37] (25,327 cells), Li *et al* [42] (20,155 cells), and Haviv *et al* [39] (9,870 cells) respectively (Supplementary Fig. 1). Further information on the datasets is in Supplementary Tables 1–2.

**Fig. 2:**
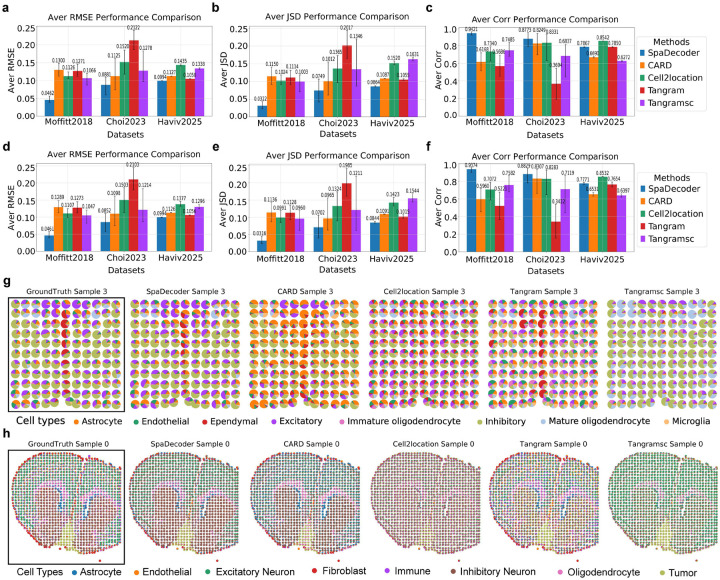
SpaDecoder qualitatively and quantitatively improves cell type deconvolution (a-f) Barplots showing the average RMSE (a,d) JSD (b,e) Pearson correlation (c,f) of SpaDecoder, CARD, Cell2location, cluster-averaged Tangram and single cell resolution Tangram (Tangramsc) across 3 single cell spatial datasets Moffitt2018, Choi2023, and Haviv2025 with simulated spots (spot size parameter $N_{\text{spot}}=50$) and simulated slice stacks $N_{\text{swap}}=2$ (a-c) and $N_{\text{swap}}=5$ (d-f). (g-h) Pie plot visualizations of cell type proportions obtained from original annotations (ground truth) in Moffitt2018 (g) and Haviv2025 (h) alongwith deconvolved outputs from SpaDecoder, CARD, Cell2location, Tangram, and Tangramsc for representative samples (sample 3) (g) and (Sample 0) (h). Each pie corresponds to a simulated spot with colors indicating the proportion of cell types in that spot.

SpaDecoder improves cell type deconvolution on all three datasets and across all three metrics with high neighboring slice similarity or fewer cell swaps per neighborhood between successive slices $\left(N_{\text{swap}}=2\right)$ (Fig. 2a–c) as well as with lower slice similarity $\left(N_{\text{swap}}=5\right)$ (Fig. 2d–f), $N_{\text{swap}}=10$ and 20 (Supplementary Fig. 2). We use moscot [33] for alignment between slices (Supplementary Fig. 3a). For an adjacent slice pair, as the distance between the slices increases, we expect each spot to be probabilistically aligned to more than one spot in an adjacent slice and therefore have a larger neighborhood. However, if adjacent slices are too dissimilar we desire that no neighborhood spots are selected from the adjacent slice. SpaDecoder's neighborhood selection strategy recapitulates this, with more simulated cell swaps between slice pairs (indicative of slice dissimilarity) leading to more neighbors selected but also resulting in an increase in the spots with no neighbors from the adjacent slice (Supplementary Fig. 3b).

Qualitative evaluation of pie plot visualizations of deconvolved cell types in each spot from the original published annotation (Ground truth), SpaDecoder, CARD, Cell2location, Tangram, and Tangramsc (Fig. 2g–h, Supplementary Fig. 1d-f) reveals that SpaDecoder most closely resembles the ground truth compared to other methods in two representative samples from Moffitt2018 (Fig 2g, Supplementary Fig. 1d), a representative sample from Haviv2025 (Fig. 2h) and two representative samples from Choi2023 (Supplementary Fig. 1e-f). In Moffitt2018, compared to other methods, SpaDecoder correctly identifies the presence of the central vertical band of ependymal cells (in red) in Sample 3 (Fig. 2g) and its absence in Sample 11 (Supplementary Fig. 1d) with lower false positive proportions of other cell types, an example of its effectiveness at capturing cell types at the interface between different cell type regions. Notably, ependymal cells are rare and comprise less than 0.24% of reference cells (Supplementary Fig. 1a). SpaDecoder also performs qualitatively better than baselines with the retina, which has a more complex tissue tissue shape (Supplementary Fig. 1e-f). Compared to other methods, pie plot visualizations qualitatively indicate that SpaDecoder distinguishes tumor (light green) from healthy tissue capturing heterogeneity in the boundary regions surrounding the tumor which is crucial for pinpointing and clinically intervening with tumor microenvironment targeted therapies (Fig. 2h).

### Ablation tests

We validate the design choices in SpaDecoder with some ablation tests. We observe that sharing information across multiple slices improves performance over single slice deconvolution (Supplementary Fig. 4). Augmenting the query spatial stack by utilizing the alignment probabilities to linearly impute gene expression at intermediate spatial locations along the z-axis also improves performance (Supplementary Fig. 5). Further, our adaptive neighborhood identification approach improves deconvolution metrics, confirming its utility (Supplementary Fig. 6). We observe a significant improvement in performance when we utilize the single cell resolution reference to learn $\Sigma_{i_{j}}$ as opposed to pseudo-bulk reference profiles (Supplementary Fig. 7). SpaDecoder also excels across varying spot size and number of cells in a spot (Supplementary Fig. 8) compared to other methods, though all methods under consideration perform worse as spot size decreases which is consistent with previous findings [22]. We also find that SpaDecoder is stable within a range of parameters for regularization λ, 3D bandwidth, and the number of slices imputed (Supplementary Fig. 9) so we keep these fixed at $\lambda =0.1,\;BW=0.01,\;N_{\text{slice}}=10$ respectively for all experiments.

### SpaDecoder decodes spot composition on multiple samples of the mouse hypothalamic preoptic region

**Fig. 3:**
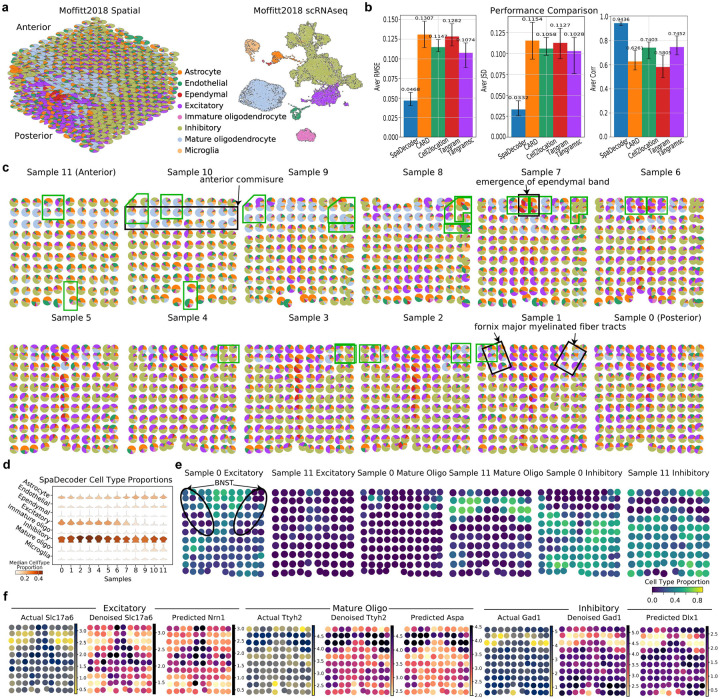
SpaDecoder captures cell type variation and expression patterns along anterior-posterior (AP) axis of the mouse hypothalamic preoptic region (a) 3D pie plots of 12 Moffitt2018 [37] samples from anterior (top) to posterior (bottom) with per-spot pies colored by ground-truth cell type proportions. Spots were simulated from MERFISH [37] (left) UMAP of scRNA-seq reference cells colored by Moffitt *et al* [37] annotations (right). (b) Barplots showing the average RMSE (left), JSD (middle), and Pearson correlation (right) between ground truth and outputs from SpaDecoder, CARD, Cell2location, cluster-averaged Tangram, and single-cell Tangram (Tangramsc). (c) Pie plots of SpaDecoder-estimated cell type proportions with overlaid green boxes indicating regions with significant composition shifts (p-value < 0.1) relative to neighboring samples, computed via Jenson-Shannon distance with within-sample permutations for p-values. The samples were divided into a 6 × 6 grid and testing was performed between corresponding regions of the grid across adjacent samples. Relevant regions in accordance with Moffitt *et al* [37] are overlaid with black boxes and labeled. (d) Stacked violin plots showing SpaDecoder cell type proportions across samples (posterior 0 → anterior 11). (e) Spatial visualizations of posterior sample 0 and anterior sample 11 with spots colored by SpaDecoder proportions of excitatory, mature oligodendrocytes and inhibitory cell types. The bed nucleus of the stria terminalis (BNST) region [37] is overlaid with a black boundary and labeled. (f) Spatial visualizations of anterior sample 11 capturing log2 normalized expression patterns of known excitatory, mature oligodendrocyte and inhibitory markers measured in the experiment (leftmost in each triplet), denoised from SpaDecoder cell spot mapping (middle in triplet), and unmeasured prediction (rightmost in triplet).

The mouse hypothalamus has been well studied due to the intricate neuronal layout, the association of specific neuronal cell types with functional roles and the likelihood of neuronal activity being somewhat conserved between mouse and human [43]. We utilize spatial data from Moffitt *et al* [37] comprising 12 evenly spaced slices or samples along the anterior to posterior axis of the mouse hypothalamic preoptic region (1.8 by 1.8 by 0.6 mm, Bregma +0.26 to −0.34) (Fig. 3a) and 25,327 scRNA-seq cells (Fig. 3a) from a similar region to evaluate the performance of SpaDecoder. We group cells into spots (Methods) and use published annotations [37] of cell types in spots as ground truth. SpaDecoder applied to the 12 samples excels quantitatively across average RMSE, correlation, and JSD, outperforming CARD, Cell2location, Tangram and Tangramsc (Fig. 3b). Qualitatively, too, cell type patterns captured by SpaDecoder from anterior (sample 11) to posterior (sample 0) more closely resemble ground truth compared to other tools (Fig. 3c, Supplementary Fig. 10). SpaDecoder recovers all reference scRNA-seq cell types namely, astrocytes, endothelial, ependymal, excitatory and inhibitory neurons, immature and mature oligodendrocytes, and microglia with varying anteroposterior proportions (Fig. 3d).

In agreement with Moffitt *et al*, SpaDecoder detects the emergence of a band of mature oligodendrocytes (light blue) in the anterior commissure which are restricted to the fornix–major myelinated fiber tracts in more posterior samples [37] (Fig. 3c labeled black boxes in samples 10 and 1, Fig. 3d–e). Conversely, immature oligodendrocytes (pink), astrocytes (orange), microglia (light orange), and endothelial (green) cells were scattered throughout the samples. Inhibitory (light green) and excitatory (purple) neurons exhibited distinct spatial patterns with the former being more widely dispersed [37] with higher proportions in specific regions toward the posterior (samples 2–4) while the latter are sparse in the anterior, generally more ubiquitous in the posterior but depleted in the posterior BNST (Fig. 3c–d, Fig. 3e labeled region). By automatically detecting regions changing in cell type composition with Jenson-Shannon distance and permutation testing, in agreement with anatomical findings [37], we are able to quantitatively pinpoint the start of the emergence of a band of ependymal cells (red cell type) beginning around sample 7 and remaining toward the posterior (Fig. 3c labeled black box in sample 7, Fig. 3d–e).

Leveraging SpaDecoder's “cell-to-cell-type association matrix” for each spot, we obtain a reference scRNA-seq to spatial mapping matrix (Methods) and use it to *denoise* the expression of measured spatial genes and *predict* unmeasured gene expression. Visualization of key cell type specific literature-driven markers [37] across excitatory (*Slc17a6*), mature oligodendrocyte (*Ttyh2*) and inhibitory (*Gad1*) cell types (Supplementary Fig. 10c) reveals high similarity between actual and denoised expression of the same gene. Correspondingly, similar patterns also arise from the prediction of unmeasured genes *Nrn1* [44], *Aspa* [45], and *Dlx1* [46] (Fig. 3f) illustrating the utility of SpaDecoder at single cell resolution spatial mapping for spatial gene expression denoising and prediction.

### SpaDecoder provides fine-grained molecular insights in human breast cancer tissue

Breast cancer can be classified into *in situ* (ductal and lobular) and invasive [47] based on localization. Ductal carcinoma in situ (DCIS) is non-invasive or pre-invasive stage 0 breast cancer and often progresses to invasive ductal carcinoma (IDC) [48]. However, the initiation of DCIS and progression to invasive is poorly understood [48]. Therefore, automatic fine grained localized spatial characterization of breast cancer tissue regions, identification of associated cell types, their spatial distribution patterns, and colocalized cell type interactions that putatively contribute to tumor progression could prove extremely beneficial.

**Fig. 4:**
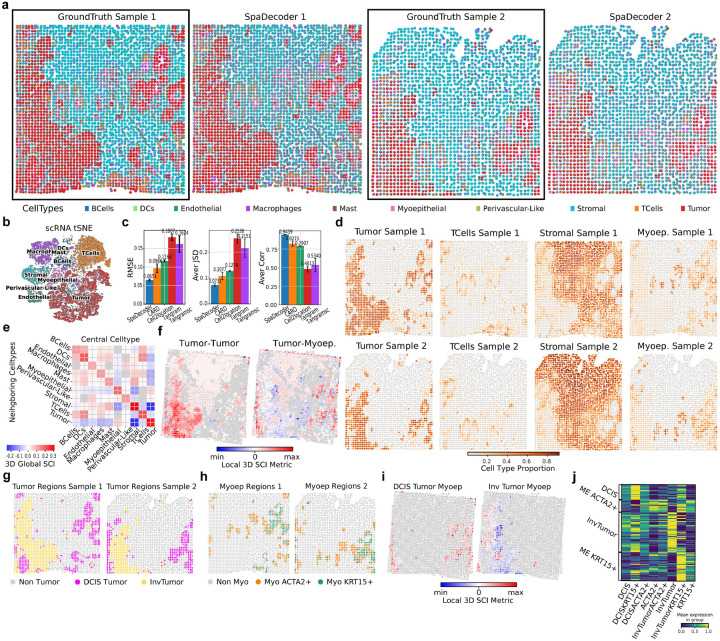
SpaDecoder decodes cell types and uncovers molecular insights in human breast cancer tissue (a) Pie plots of cell type proportions from ground-truth annotations (Janesick *et al* [40], black box) and SpaDecoder on two Xenium samples with neighborhood-aggregated spots. (b) tSNE embedding of scRNA-seq reference cells (Janesick *et al* [40]), colored by cell type. (c) Barplots showing average RMSE, JSD, and Pearson correlation between ground truth and deconvolved proportions from SpaDecoder, CARD, Cell2location, Tangram, and Tangramsc. (d) Spatial plots of sample 1 and 2 with spots colored by SpaDecoder proportions of key cell types (Tumor, T, Stromal, and Myoepithelial (ME)). (e) Heatmap of global averaged 3D local neighborhood colocalization between cell type pairs (central type: X-axis; neighbor type: Y-axis). Negative values indicate neighboring type depletion, positive values enrichment; nonsignificant pairs (p > 0.05) in gray. Significance is obtained via permutation testing (Methods). (f) Aligned slices showing 3D local colocalization for Tumor-Tumor and Tumor-Myoepithelial (ME) cell type pairs. The first in the pair indicates the central cell type while the second is the neighborhood cell type. Negative values indicate neighboring type depletion, positive values enrichment; nonsignificant (p-value > 0.05) in gray. (g) Spatial slices colored by tumor subtypes identified from tumor regions obtained from connected component analysis of SpaDecoder tumor cell type proportions. (h) Spatial slices colored by ME (Myoep) subtypes identified from ME regions obtained from connected component analysis of SpaDecoder ME cell type proportions. (i) Aligned samples showing 3D local colocalization for DCIS–ME (Myoep), and invasive tumor–ME (Myoep); nonsignificant spots (p-value > 0.05) in gray. (j) Heatmap of scaled mean expression of data-driven, cell type–enriched scRNA-seq markers (Y-axis) across tumor and ME subtype spatial regions visualized in Supplementary Fig. 11f.

Here, we apply SpaDecoder to two 5μm breast cancer tissue samples or slices (Fig. 4a) along with a reference scRNAseq dataset (Fig. 4b) comprising 26, 031 cells from Janesick *et al* [40]. The spatial samples were profiled at single cell resolution with 10x Xenium and 3201 and 2199 spots were obtained respectively using our spot generation procedure (Methods). Ground truth cell type proportions for the spots were obtained from given annotations in Janesick *et al* [40]. SpaDecoder identifies B, dendritic (DCs), Endothelial, Macrophages, Mast, Myoepithelial (ME), Perivascular-like, Stromal, T, and Tumor cells in both samples with patterns resembling the corresponding sample ground truth according to the original published annotations (Fig. 4a). Qualitatively, our identified cell type patterns more closely resemble ground truth compared to CARD, Cell2location, Tangram and Tangramsc (Fig. 4a, Supplementary Fig. 11a-d) and quantitative evaluation across Average RMSE, Pearson Correlation, and JSD confirms SpaDecoder's superior performance over baseline methods (Fig. 4c). Visualizing the proportions of the key cell types (tumor, stromal, T, and ME) revealed spatial patterns in the cell type distribution (Fig. 4d). In particular, consistent with Janesick *et al* [40], we observe a large mostly connected region of stromal cells, several groups of tumor cells on left and right sides with different spatial shapes, and some less pronounced grouping of T and ME cells.

We then investigated if any of the cell types had strong colocalization. Utilizing the SpaDecoder cell type proportions, we computed our Global 3D Cell Type Spatial Colocalization Index (G-3DSCI) (Methods) (Fig. 4e). We observe that tumor cells are anti-colocalized with the broadly grouped stromal cells but positively colocalized with other tumor and ME cells (Fig. 4d–e). This means that compared to a random 3D neighborhood, on average, tumor cells are more likely to occur in the immediate vicinity of other tumor and ME cells and less likely to co-occur with healthy stromal cells. The colocalization of ME and tumor cells is interesting since the former have been previously shown to play a critical role of tumor progression [49]. We therefore sought to localize spots where tumor cells were surrounded by neighboring cells significantly (pval<0.01) enriched or depleted in ME cells. To this end, we used our Local 3D Cell Type Spatial Colocalization Index (L-3DSCI) metric (Methods) which tested the colocalization in the neighborhood surrounding each spot compared to a randomly chosen background. The results revealed individual spots with neighborhoods of colocalization of tumor cells with each other, and tumor cells with ME cells (Fig. 4f).

Since tumor cells often group together in specific patterns and are surrounded by other cell types, we performed Cell Type Region Identification (CTRI) to spatially localize contiguous tissue regions containing cells of a particular type with connected cell component analysis (Methods) and identified T, tumor and ME regions (Supplementary Fig. 11e). Leveraging fine grained annotations from Janesick *et al* [40] and our single cell to spatial mapping, we further classify tumor and ME regions into their respective cell subtypes using “cell subtype identification in regions” (Methods) and identify DCIS tumor, Invasive tumor, ACTA2+ ME, and KRT15+ ME cells (Fig. 4g–h, Supplementary Fig. 11f). We observe a distinct pattern of invasive cells to the left in both samples while DCIS cells occur on the left and right but have a more pronounced oval shape toward the right, consistent with Janesick *et al* [40]. Further, unlike on the left where ME cells are fewer and less structured, ME cells on the right form roughly concentric ME KRT15+ (inner) and ME ACTA2+ (outer) patterns. We note the existence of ACTA2+ regions without the presence of KRT15+ regions, a pattern also observed in Janesick *et al*. It also appears that DCIS colocalizes with ME, which is confirmed by L-3DSCI (Methods) analysis showing significant positive colocalization between DCIS and ME with almost no negative colocalization on both left and right sides of the samples (Fig. 4i). Additionally, we observe few positively and largely negatively colocalized Invasive tumor and ME cell spot neighborhoods (Fig. 4i). This indicates a significant presence of ME cells in DCIS neighborhoods and an absence in most invasive neighborhoods, consistent with the hypothesis that ME cells act as a barrier and loss of ME cells and their associated basement membrane is a hallmark of the transition from DCIS to IDC [49]. We confirmed our DCIS, invasive, ACTA2+ ME, KRT15+ ME cell subtype annotations by identifying differentially expressed genes in the corresponding categories from the reference scRNA-seq dataset (adjusted p-val < 0.05, log2FC > 1.0) and visualizing their expression on the spots with annotated subtypes or pairs of subtypes with good consistency (Fig. 4j).

In conclusion, SpaDecoder qualitatively and quantitively proved successful in identifying cell types. Utilizing SpaDecoder outputs, we performed downstream analyses and identified cell subtypes, uncovered cell type occurence and colocalization patterns globally as well as at spot resolution. Further, we identified putatively interesting regions of tumor and ME cooccurence, annotated their subtypes and provided evidence supporting the hypothesis that ME cells act as a barrier to the transition from DCIS to invasive tumor [49].

### SpaDecoder reveals spatio-temporal cell type organization during human fetal thymic development

SpaDecoder has demonstrated excellent performance across various tissues and technologies by sharing information across 3D spatial tissue slices. We next inquire if SpaDecoder can perform well in the spatio-temporal scenario, such as during organ development when 2D tissue slices are profiled across time. We sought a recently published spatial atlas capturing human fetal and pediatric thymic development [41] and selected the longest continuous range of spatially profiled time points comprising four 10x Visium samples spanning 16 to 19 post conception weeks (pcw) sampled weekly and scRNA-seq data at 15pcw, 17pcw, and 18pcw.

The thymus is a specialized organ of the immune system responsible for T Cell maturation and education [50]. It has two lobes joined by connective tissue and surrounded by a connective tissue capsule with septae or partitions that extend inward from the capsule [41]. Internally, it has two distinct compartments, the outer dense cortex and inner medulla, along with secondary structures such as medullary Hassall's corpuscles. [41]. Tcell maturation which begins as early as 8 post conception weeks (pcw) in humans is a highly organized and regulated process [51]. Developing Tcells are broadly categorized based on their expression of CD4 and CD8 into CD4−CD8− Double negative (DN), CD4+CD8+ double positive (DP), or single positive (SP) (either CD4+CD8− or CD4−CD8+) [52]. Early Tcell Progenitors (ETPs) enter the thymic medulla around the corticomedullary junction (CMJ) and migrate to the cortex where they may undergo maturation to DN and SP through interaction with cortical thymic epithelial cells (cTECs), differentiate into CD4+ and CD8+ T cell lineages via positive selection and migrate to the medulla [53].

**Fig. 5:**
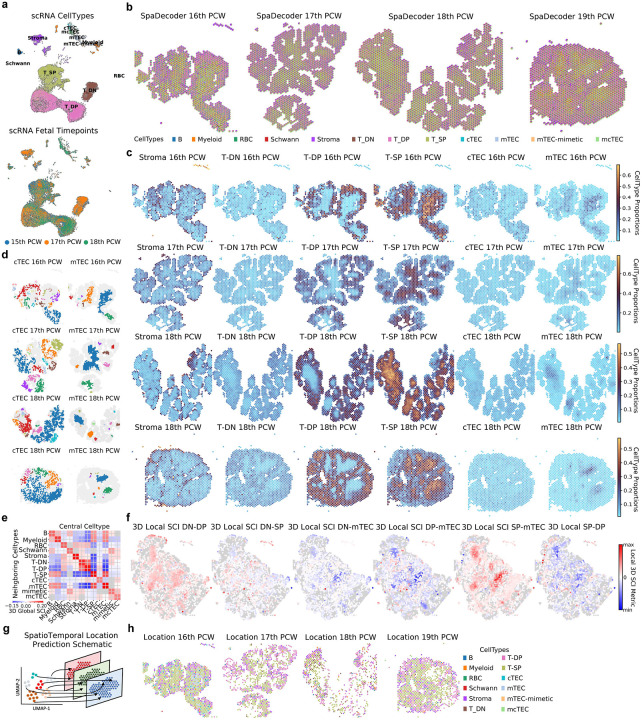
SpaDecoder decodes spatiotemporal tissue organization during human fetal thymic development (a) UMAP of scRNA-seq reference cells (Yayon *et al* [41]) colored by cell type (top) and fetal timepoint (bottom). (b) Pie plots of SpaDecoder-inferred cell type proportions on Visium slices or samples from 16–19PCW of human fetal thymic development. (c) Spatial plots showing SpaDecoder proportions of key cell types (Stroma, DN-T, DP-T, SP-T, cTEC, mTEC). (d) cTEC and mTEC regions identified at each time point via connected component analysis. (e) Heatmap of averaged 3D local neighborhood colocalization between cell type pairs (central type: X-axis; neighbor type: Y-axis); negative values indicate depletion, positive enrichment; nonsignificant pairs (p> 0.05) in gray. (f) Aligned samples showing 3D local colocalization for DN-DP, DN-SP, DN-mTEC, DP-mTEC, SP-mTEC, and SP-DP; nonsignificant spots (p>0.05) in gray. (g) Schematic of SpaDecoder-inferred cell-to-spot mapping matrix predicting single-cell spatiotemporal coordinates. (h) Predicted spatial coordinates of reference scRNA-seq cells across timepoints colored by cell type.

After excluding ambiguous annotations, we were left with 34,798 cells visualized on the published UMAP embedding (Fig. 5a) colored by annotated cell type (top) and developmental day (bottom) [41]. We selected an scRNA-seq annotation resolution that would capture the heterogeneity in major cell types but was not overly fine-grained and indiscernible. In particular we annotated Schwann cells, red blood cells (RBCs), myeloid, cTECs, mTECs, mcTECs (putative bipotent TEC progenitor cells), mimetics, DN, DP, and SP cells from the original published annotations [41] (Fig. 5a, top). Despite the variability in spatial tissue shape across the samples, SpaDecoder identified all the cell types (Fig. 5b) qualitatively, based on visual similarity to known thymic structure described above, outperforming Cell2location and Tangramsc (Supplementary Fig. 12). Visualization of deconvolution proportions on the spatial embeddings at each of the time points revealed key regions. At each time point, consistent with thymic structure, we identify stromal cells in the capsule and along the septae (Fig. 5c). DN cells are in lower proportions as expected but concentrated around the periphery consistent with their presence in the cortex. DP cells are much more prominent than DN but also largely in the cortex, unlike SP cells which are highly concentrated in the interior medulla. As expected, the thymus is overrun with developing T-lymphocytes and TECs are much less abundant (Fig. 5c). Therefore, to further delineate mTEC and cTEC regions, we performed CTRI (Methods) and found that while cTECs are present in the outer cortex, the mTECs are in higher proportion with more clearly demarcated localization patterns in the inner medulla (Fig. 5c–d). Interestingly, the cortex is subdivided into more regions than the medulla which could be due to the septae that extend inward from the capsule partially fragmenting it (Fig. 5c–d). Despite the fact that the two thymic lobes are clearly demarcated at 16 and 19pcw while they are less clear in the 17–18pcw samples likely due to technical differences when processing, SpaDecoder is able to capture the cell types effectively (Fig. 5b–d).

Consistent with the described structure of the thymus, global and local cell type colocalization revealed positive spatial colocalization between DN and DP in the cortex, and between SP and mTECs which are both medullary (Fig. 5e–f). We also observed negative spatial colocalization between DN (cortical) and SP (medullary), DN and mTEC, between DP and mTECs, and between SP (medullary) and DP (cortical) (Fig. 5e–f), consistent with thymic structure. Finally, we recapitulate the spatio-temporal cell layout by mapping each scRNA-seq cell to the spot across time and space with the maximum mapping probability (Fig. 5g) and observe that the recovered spatial patterns closely resemble the original spatial layout at each time point (Fig. 5h).

## Discussion

The surge of spot based spatial transcriptomics (ST) from stacks of 3D spatial tissue slices or across developmental or disease timecourses, motivates the need for developing deconvolution algorithms that can leverage the 3D spatiotemporal tissue structure to decode cell types and elucidate underlying molecular processes, while exploiting vast available scRNA-seq atlases. Here, we present SpaDecoder, a computational model for ST spot deconvolution, designed for a 3D stack of multiple spatial or temporal tissue slices.

While myriad methods exist for single 2D ST tissue slice deconvolution, the design of SpaDecoder overcomes several limitations and presents directions for research in other 3D ST problems, such as spatially variable gene identification and spatial domain identification. Overall, SpaDecoder aligns spatiotemporal slices with moscot [33] and imputes additional intermediate slices for data augmentation, which improves performance. It further infers spatially and transcriptionally similar 3D neighbors for each spot, by defining the LSTA metric and performing permutation testing. Notably, this does not rely on the assumption that spatially neighboring spots are similar, which can be violated in more complex heterogeneous tissue environments [36]. It then defines a spatiotemporal Gaussian kernel on the estimated neighborhood to leverage 3D tissue structure. SpaDecoder also benefits from cellular resolution of the scRNA-seq reference, decomposing spot expression into the product of reference scRNA-seq expression, a cell-to-cell type association matrix and the spot deconvolution proportions, thus capturing subtle cell state variation within cells of the same type.

The mathematical framework of SpaDecoder enables its use in a range of downstream applications. We define the G-3DSCI metric and perform permutation testing to reveal colocalized cell types across all the slices. To refine our observations to individual neighborhoods with colocalization, we further define L-3DSCI. We identify tissue regions changing cell type composition across the 3D slice stack with a Jenson-Shannon distance metric and permutation testing. We also identify contiguous cell type regions within a tissue with connected component analysis. These defined metrics and applications have broad applicability across tissue type, as they leverage cell type proportions and can be combined with other deconvolution methods. SpaDecoder's spot expression decomposition objective function also enables mapping of reference scRNA-seq cells to spatial spots, enabling fine grained cell type annotation transfer, as well as the imputation of unmeasured genes in 3D space. Notably, the mapping also enables the prediction of 3D spatio-temporal locations of scRNA-seq cells.

We extensively evaluate the performance SpaDecoder in several scenarios. To overcome the lack of ground truth, we first utilize simulated spots from single cell spatial transcriptomic datasets and simulate tissue slice stacks by perturbing a single slice. We present quantitative results with established evaluation metrics (Root Mean Square Error, Jenson-Shannon Divergence, Pearson correlation) utilizing the original published annotations from spatial single cells as the ground truth. SpaDecoder excels with varying spot sizes and slice stack simulation conditions. SpaDecoder is stable within a range of hyperparameter choices. Ablation tests reveal improvement with each component of our method - the use of single cell resolution reference scRNA-seq data, multiple spatial slices, intermediate slice imputation for data augmentation, and adaptive neighborhood selection.

SpaDecoder qualitatively and quantitatively improved deconvolution, compared to baseline methods, on 12 anterior-posterior (A-P) MERFISH samples of the mouse hypothalamic preoptic region from Moffitt *et al* [37]. We identify cell types with characteristic spatial organization patterns, and uncover regions that gradually change composition along the AP axis in 3D stack. We map scRNA-seq cells to spatial spots enabling imputation of unmeasured genes associated with key cell types. On 2 breast cancer samples profiled by Janesick *et al* [40], SpaDecoder identifies distinct tumor, stromal, T and myoepithelial (ME) cell patterns, and spatially colocalized cell types across 3D. We categorize tumor regions into ductal carcinoma *in situ* (DCIS) and invasive, and ME regions into ACTA2+ and KRT15+. Our analyses supports the hypothesis that ME cells act as a barrier for progression from DCIS to invasive tumor [49]. SpaDecoder additionally excels on a 3D spatiotemporal human fetal spatial thymic atlas profiled with 10x Visium capturing 16pcw-19pcw sampled weekly with a reference scRNA-seq atlas across 15pcw, 17pcw, and 18pcw. Despite the technical variations in tissue shape, SpaDecoder performed well, capturing cell types, global and local colocalization patterns, and regions according to their known occurence in the cortical and medullary thymic regions. Importantly, SpaDecoder spatio-temporally mapped scRNA-seq cells to spatial spots showing broad application potential to spatially profiled developmental and disease time courses.

Overall, SpaDecoder qualitatively and quantitatively outperforms other benchmarked methods on various metrics, datasets, scenarios, and ablations, and yields interpretable biology, showing it effectively harnesses 3D tissue structure and single cell reference profiles to improve deconvolution. We anticipate that SpaDecoder, as one of the earliest methods leveraging 3D tissue structure [34, 54], will motivate an increase in the number of tools that utilize 3D ST data for various computational tasks. We envisage broad adoption of our defined metrics and application scenarios by experimentalists in diverse problem scenarios.

## Methods

Given multi-slice spot-based spatial transcriptomic data, the computational pipeline of SpaDecoder is illustrated in Figs. 1a–f. After preprocessing the input data, SpaDecoder aligns spots between spatially or temporally adjacent slices using Optimal Transport and augments the data by imputing intermediate slices between adjacent slices. SpaDecoder then infers the neighborhood for each spot by defining a *local spatially weighted transcriptomic autocorrelation* metric (LSTA) and performing permutation testing to identify the largest neighborhood with spatio-transcriptomic similarity, thus adapting to local tissue heterogeneity. SpaDecoder uses a 3D spatial Gaussian kernel in this neighborhood to model spot similarity, within each slice and across original and imputed slices. With this adaptive kernel, SpaDecoder formulates a multi-slice objective function for each target spot. It factorizes the gene expression with the reference scRNA-seq expression, a reference cell-to-cell-type association matrix, and the proportion of each cell type in the target spot. The SpaDecoder loss minimizes the 3D kernel weighted distance between the factorized gene expression of the current spot and the expression of neighboring spots in the 3D tissue space. By optimizing this objective, for each spot SpaDecoder estimates the cell type proportions and the scRNA-seq cell-to-cell-type association matrix which can be leveraged for diverse downstream applications in spatial cell type analysis.

In the following sections we describe SpaDecoder in detail. First, we explain SpaDecoder preprocessing which includes data preprocessing, slice alignment and imputation, adaptive spatio-transcriptomic neighborhood selection and kernel estimation. Next, we describe the formulation of the multi-slice objective function and optimization of SpaDecoder. We detail the downstream applications of SpaDecoder and describe the datasets and simulations used. Finally, we explain the running of the baseline methods and evaluation metrics in the benchmark.

### SpaDecoder Preprocessing

#### Data Preprocessing

We apply standard preprocessing corresponding to best practices for handling scRNA-seq and spatial datasets [55]. Raw scRNA-seq count matrices were obtained from the respective datasets (described in Data section below) and standard pre-processing was performed using scanpy [56]. Genes present in less than 10 cells were excluded and the subset of overlapping genes between scRNA-seq and spatial data was selected. Cells with minimum total counts < 50 were excluded. In case there were over 5000 resulting genes in the set, we performed additional gene feature selection. For scRNA-seq data, we normalize (sc.pp.normalize_total) and log pseudo count transform (sc.pp.log1p) the count matrix, and select the differentially upregulated genes in each defined scRNA-seq cluster (adjusted p-value < 0.05, log-foldchange > 1.0). We concatenate all spatial slices and select the top 5000 slice-aware highly variable genes (sc.pp.highly_variable_genes) to avoid the selection of slice specific genes. The overlap between spatial highly variable genes and scRNA-seq differentially upregulated genes comprises the resulting feature set.

scRNA-seq and spatial data is normalized using sc.pp.normalize_total with target_sum = 1000 to correct for varying sequencing depth. Since different spatial datasets might have different coordinates and spatial distances between spots, we scale X-Y spatial coordinates to [0, 1].

#### Slice Alignment

We align corresponding spots across different slices with moscot [33], an optimal-transport-based algorithm (Fig. 1e). Moscot [33] improves on PASTE [4] and outputs a coupling matrix P between each pair of slices where $P_{ij}$ denotes the probability mass transported from spot i in the left slice to spot j in the right slice. It uses the balanced entropic regularized Fused-Gromov Wasserstein Optimal Transport (FGW-OT) formulation to minimize the transcriptomic distance between mapped spots across the two slices as well as maximizes the correspondence between spatial arrangement of spots for each slice with its immediate neighbors. For two slices X and Y, let a denote the set of spots in X and b the corresponding set in Y, then the general form of an FGW-OT problem is

(1) $$P^{*}≔{\text{argmin}}_{P\in U(a,b)}\;\alpha \sum_{i,j,i^{\prime },j^{\prime }}L\left(C_{\text{ij}}^{X},C_{i^{\prime }j^{\prime }}^{Y}\right)P_{ii^{\prime }}P_{jj^{\prime }}+(1-\alpha )\sum_{ii^{\prime }}C_{ii^{\prime }}P_{ii^{\prime }}-\varepsilon H(P)\;\text{where}\;H(P)≔-\sum_{i,i^{\prime }}\;P_{i,i^{\prime }}\left(\text{log}\left(P_{i,i^{\prime }}\right)-1\right)$$

for spots i,j∈X and $i^{\prime },\;j^{\prime }\in Y$ where the first GW term quantifies the spatial correspondence between the slices, the second W term represents transcriptomic matching with α controlling the weight between them and H(P) is the entropy regularization term with strength ε. To obtain the matrix C, the normalized preprocessed counts from both slices are projected onto a joint PCA space (with 30 components by default) and the squared Euclidean distance is computed between pairs of spots from the two slices. The matrices $C^{X}$ and $C^{Y}$ are obtained from the squared Euclidean distance on normalized spatial coordinates in slice X and Y respectively. $L\left(C_{ij}^{X},C_{i^{\prime }j^{\prime }}^{Y}\right)={\left(C_{ij}^{X}-C_{i^{\prime }j^{\prime }}^{Y}\right)}^{2}$. In SpaDecoder, we set α=0.5 for all the experiments. The full rank solver from Peyré et al [57] as implemented in moscot [33] was used.

#### Intermediate Slice Imputation

We augment the spatial data through intermediate slice imputation to improve the inference accuracy of SpaDecoder(Fig. 1e). For each adjacent slice pair in the input dataset, we simulate $N_{imp}$ slices. Let $P_{p_{q}i_{j}}$ be the alignment probability between the jth spot in slice i (current slice) and qth spot in slice p (adjacent slice) obtained from slice alignment. Let $t=1,2,\cdots ,N_{\text{imp}}-1$ be the slices to impute. $x_{i_{j}},x_{p_{q}}$ are the G×1 expression vectors of the jth spot in slice i and the qth spot in slice p respectively. G is the number of selected genes. $N_{p}$ is the number of spots in slice p.𝒩(μ,Σ) represents Gaussian noise with mean μ and standard deviation Σ. Then we perform linear interpolation to obtain expression vector $x_{t_{j}}$ for spot j in intermediate slice t. Slice t is of the same shape as slice i.

(2) $$x_{i_{j}t_{p_{q}}}=x_{p_{q}}+\frac{t}{T}\left(x_{i_{j}}-x_{p_{q}}\right)+𝒩(0,0.1)\;x_{t_{j}}={\left(x_{i_{j}t_{p:}}\right)}_{G\times N_{p}}\times {\left(P_{p_{:}i_{j}}\right)}_{N_{p}\times 1}$$:

denotes all the spots in slice p. We set $N_{imp}=20$ by default but observe stable performance between 10 and 20 (Supplementary Fig. 9).

#### Adaptive Neighborhood Selection and Kernel Estimation

For each spot j in slice i, SpaDecoder adaptively infers a neighborhood $nbd_{i_{j}}$ comprising transcriptionally similar spatial neighbors which it uses to define a 3D spatial Gaussian kernel. Let ${\left(D_{i_{j}p_{q}}\right)}_{spa}$ be the spatial distance between the jth spot in the ith slice and the qth spot in the pth slice. For spots in the same slice, i=p, we define ${\left(D_{i_{j}i_{q}}\right)}_{\text{spa}}$ as the squared Euclidean distance between X-Y spatial coordinates of the two spots. Otherwise, ${\left(D_{i_{j}p_{q}}\right)}_{spa}$ is derived from the alignment probability matrix $P^{*}$ (1). In this case, ${\left(D_{i_{j}p_{q}}\right)}_{spa}$ may not be symmetric since the number of spots in the two slices may be different. Let slice i,p have $N_{i},\;N_{p}$ spots with i being the current slice and p the neighboring slice. Then, we scale the transport matrix $P_{i_{j}p_{q}}^{*}/\text{max}(P_{i_{j}p_{q}}^{*})$, clip low transport probabilities at 0.00001, convert it into a negative log distance and rescale by the maximum value to get ${\left(D_{i_{j}p_{q}}\right)}_{spa}$. We then perform filtering $\left({\left(D_{i_{j}p_{q}}\right)}_{spa}\geq 1e^{-}7\right)$, normalization ${\left(D_{i_{j}p_{q}}\right)}_{spa}/\text{max}({\left(D_{i_{j}p_{q}}\right)}_{spa})$ and then define an X-Y Gaussian kernel to model the weighted spatial distances between any two spots:

(3) $$W_{i_{j}p_{q}}^{\text{Gaussian}}=\text{exp}\left\{-\frac{1}{2}\left(\frac{{\left(D_{i_{j}p_{q}}\right)}_{\text{spa}}}{C}\right)\right\}\;C=0.5\times \text{BW}\times \text{Med}\;W_{i_{j}p_{q}}^{X-Y^{t}}=W_{i_{j}p_{q}}^{\text{Gaussian}}M_{i_{j}p_{q}}^{t}\;\text{where}\;M_{i_{j}p_{q}}^{t}=\left\{\begin{array}{ll} 1 & \text{if}\;\text{spot}\;p_{q}\in nb{d_{i_{j}}}^{t}\\ 0 & \text{otherwise} \end{array}\right.$$

C is a constant determined by the hyperparameter bandwidth BW and Med is the median ${\left(D_{i_{j}p_{q}}\right)}_{spa}$ in the slice. We define $t_{\text{max}}$ binary masks $M^{t}$ for each spot, $t=1\cdots t_{\text{max}}$ by selecting the top t transcriptomic neighbors as measured by squared Euclidean transcriptomic distance ${(D}_{i_{j}p_{q}}^{t})$ and setting low distance $<1e^{-10}$ to 0. If a spot lies in the neighborhood of another spot the mask is 1, else 0. We consider a spot to be a neighbor of itself and choose $t_{\text{max}}=30$.

Spatially proximal spots may not always have similar transcriptomic profiles, particularly in complex heterogeneous tissue structures or at the boundary between cellular structures [36]. This is also tissue, region and even spot dependent since we would benefit from using more neighbors in more homogeneous tissue environments compared to fewer neighbors in heterogeneous regions. Hence, we define a *local spatially weighted transcriptomic autocorrelation* metric (LSTA) and perform permutation testing to select the spatial neighborhood with high transcriptomic similarity for each spot $i_{j}$ and mask $M^{t}$:

(4) $$LST{A^{t}}_{i_{j}}=\sum_{p_{q}\in nbd_{i_{j}}t}\;W_{i_{j}p_{q}}^{t}D_{i_{j}p_{q}}^{t}$$

LSTA is an adaptation of the local multivariate GearyC metric [58]. Lower LSTA values indicate lower weighted transcriptomic distances and therefore are a good choice for neighborhood. We perform permutation testing for each spot, by permuting all other spots, and computing LSTAN=500 times to obtain a p-value and restrict to significant neighborhoods (p-value < 0.05). To maximize the number of data points available, we select the kernel from the significant neighborhood with the largest number of neighbors as the X-Y kernel $W_{i_{j}p_{q}}^{X-Y}$. If no neighborhood has significant LSTA, the spot may lie in a highly heterogeneous region, so we define the neighborhood to be the spot itself. This operation is parallelized across all spots, slices, and permutations, with optional selection of batch size, thereby optimizing runtime. For spot j in an augmented slice t between a current i and neighboring slice p, the 2D kernel weights are $W_{i_{j}p_{q}}^{X-Y}$ since the X-Y spatial locations do not change with augmentation in our approach, with variation captured across slices along the z-axis.

We further utilize a Gaussian kernel to account for the spatio-temporal variation along the z-axis. We index the real and augmented slices in the stack. For real or augmented slice with index p and selected slice with index i:

(5) $$W_{ip}^{Z}=\frac{1}{\sigma \sqrt{2\pi }}\text{exp}\frac{-{\overset{‾}{z}}^{2}}{2\sigma^{2}}$$

where $\overset{‾}{z}=|i-p|$ is the slice gap and $\sigma =BW_{3D}/4$ where $BW_{3D}$ is the 3D bandwidth hyperparameter. We set $BW_{3D}=8$, though results are stable in 8 to 16 (Supplementary Fig. 9). The resulting kernel in 8 is the product of the X-Y and Z kernels:

(6) $$w_{i_{j}p_{q}}=W_{i_{j}p_{q}}^{X-Y}\cdot W_{ip}^{Z}$$

### Multi-slice Objective Function

Given the count matrix of the spatial transcriptomic dataset X and scRNA-seq dataset $B_{sc}$, for G selected gene features, SpaDecoder decomposes the gene expression $x_{i_{j}}$ of spot j in slice i (spot $i_{j}$) into $B_{sc}{𝚺}_{i_{j}}V_{i_{j}}$ where ${𝚺}_{i_{j}}$ is the scRNA-seq cell-to-cell-type association matrix and $V_{i_{j}}$ is the deconvolution vector containing proportions for each annotated scRNA-seq reference cell type at each spatial spot j in every slice i. For a learnable spot correction scalar $\alpha_{i_{j}}$, following the Mean Square Error (MSE) objective:

(7) $$\alpha_{i_{j}}^{⋆},{𝚺}_{i_{j}}^{⋆},{\tilde{V}}_{i_{j}}^{⋆}=\text{arg}\underset{\alpha_{i_{j}},{𝚺}_{i_{j}},{\tilde{V}}_{i_{j}}}{\text{min}}\;\frac{1}{G}{\left\|x_{i_{j}}-\alpha_{i_{j}}B_{sc}{𝚺}_{i_{j}}V_{i_{j}}\right\|}_{2}^{2}$$

To capture subtle cell-state variability among cells of the same type, ${𝚺}_{i_{j}}$ is initialized with normalized cell type annotation indicator variables, relaxed onto the simplex via the softmax function, thus learning soft cell-to-cell-type assignment. $V_{i_{j}}$ is also constrained to the probability simplex using softmax, modeling cell type proportions in each spot. To penalize large pre-constrained $V_{i_{j}}$, the L2-regularization term $\lambda {\left\|V_{i_{j}}\right\|}_{2}^{2}$ is added.

When deconvolving spot j in slice i, to further utilize 3D spatial information, SpaDecoder considers not only the expression value $x_{i_{j}}$ of spot $i_{j}$, but also the expression values $x_{p_{q}}$, for all spots q in slice p in the 3D spatio-transcriptomic neighborhood $nbd_{i_{j}}$ of spot $i_{j}$. Each spot can have variable number of neighbors depending on whether it is located in a homogeneous or heterogeneous region (see “Adaptive Neighborhood Selection and Kernel Estimation” section). The final objective function is the weighted sum considering all $i_{j}$'s transcriptionally similar spatially proximal spots $p_{q}$, with the weight $w_{i_{j}p_{q}}$ representing the 3D similarity between spot $i_{j}$ and spot $p_{q}$.


(8) $$\alpha_{i_{j}}^{⋆},{𝚺}_{i_{j}}^{⋆},{\tilde{V}}_{i_{j}}^{⋆}=\text{arg}\underset{\alpha_{i_{j}},{𝚺}_{i_{j}},{\tilde{V}}_{i_{j}}}{\text{min}}\;\frac{1}{G}\sum_{p_{q}\in nbd_{i_{j}}}\;\;w_{i_{j}p_{q}}{\left\|x_{p_{q}}-\alpha_{i_{j}}B_{sc}{𝚺}_{i_{j}}V_{i_{j}}\right\|}_{2}^{2}+\lambda {\left\|{\tilde{V}}_{i_{j}}\right\|}_{2}^{2}\;\text{s.t.}\;{𝚺}_{i_{j}}\geq 0,\;{𝚺}_{i_{j}}^{T}1=1,\;\alpha_{i_{j}}\geq \epsilon \;\text{where}\;V_{i_{j}}=\Pi_{\Delta }\left({\tilde{V}}_{i_{j}}\right),\;\text{and}\;\Delta =\left\{V|V\geq 0,\;V^{T}1=1\right\}.$$

Since each spot is optimized separately, we speed up runtime by computing in parallel on a GPU the deconvolution scores for all (or optionally for a fixed user provided batch size) spots in the 3D tissue. For each spot $i_{j}$, the final output of SpaDecoder comprises the cell type deconvolution scores $V_{i_{j}}$, cell-to-cell-type association matrix ${𝚺}_{i_{j}}$ and scalar $\alpha_{i_{j}}$. $B_{sc}{𝚺}_{i_{j}}v_{i_{j}}$ yields the imputed gene expression of spot $i_{j}$ and ${𝚺}_{i_{j}}v_{i_{j}}$ maps scRNA-seq cells to spot $i_{j}$, enabling fine grained spatial cell type annotation and the prediction of spatial locations of scRNA-seq cells. We use these for downstream analyses (details in “Applications of SpaDecoder” section).

### Optimization of SpaDecoder

All SpaDecoder experiments (except for testing parameter effects) are run with default parameters. Regularization parameter λ=0.1. Adam optimization was performed with 500 iterations, early stopping if the cost function changes by < 10^−4^ between consecutive iterations and learning rate 0.01. In SpaDecoder-cluster (Supplementary Fig. 4), the cluster averaged version of SpaDecoder, each spot was initialized with cell type proportions from the reference. To prevent SpaDecoder from getting stuck in a local minima, deconvolution proportions for each spot were initialized with SpaDecoder-Cluster output. The scalar spot parameter *alpha* was initialized to 1. SpaDecoder was run in batch mode on an A40 GPU so multiple 3D spots could be processed in parallel. Permutation testing for the LSTA metric was also parallelized.

### Applications of SpaDecoder

We define several metrics from the SpaDecoder outputs and use them in downstream analyses for biological insights (Fig. 1g–h).

#### Global 3D Cell Type Spatial Colocalization Index (3DC-SCI)

Utilizing the SpaDecoder cell type proportions, we developed a 3D spatial cell colocalization metric motivated by the 2D global gene spatial cross-correlation index (SCI) in Meringue [59] (Fig. 1g). For every slice, we stack the slice before and after (except for the first and last slice which is a stack of 2), align them, and project the X-Y coordinates onto the center slice with moscot [33]. For the z-coordinate, use the the position in the tissue or an arbitrary index which preserves the ordering. We obtain the 3D spatial neighbors with Delaunay triangulation [60] and ensure that a spot is a neighbor to itself since a spot may comprise > 1 cell type. Then we compute 3DC-SCI for cell types x and y as:

(9) $$\text{3DC-SCI}(x,y)=\frac{N}{2\sum_{i=1}^{N}\sum_{j=1}^{N}w_{ij}}\cdot \frac{\sum_{i=1}^{N}\sum_{j=1}^{N}w_{ij}\left(x_{i}-\overset{‾}{x}\right)\left(y_{j}-\overset{‾}{y}\right)}{\sqrt{\left(\sum_{i=1}^{N}{\left(x_{i}-\overset{‾}{x}\right)}^{2}\right)\left(\sum_{j=1}^{N}{\left(y_{j}-\overset{‾}{y}\right)}^{2}\right)}}$$

where N is the number of spots, $W_{ij}$ is the binarized spatial connectivity matrix, $x_{i}$ and $y_{j}$ are the cell type proportions of cell type x and y at spot i and j and $\overset{‾}{x}$ represents the mean cell type proportion across the stack. Next, we recompute the metric after permuting the spots P=1000 times to obtain a p-value.

#### Local 3D Cell Type Spatial Colocalization Index (L3DC-SCI)

Similar to 3DC-SCI we define L3DC-SCI as the localized cross correlation between the cell type proportion in the center spot and its neighbors as follows:

(10) $$\text{L3DC}–\text{SCI}(x,y)=\frac{N}{\sum_{i=1}^{N}\;\sum_{j=1}^{N}w_{ij}}\cdot \frac{\left(x_{i}-\overset{‾}{x}\right)\sum_{j=1}^{N}w_{ij}\left(y_{j}-\overset{‾}{y}\right)}{\sqrt{\left(\sum_{i=1}^{N}{\left(x_{i}-\overset{‾}{x}\right)}^{2}\right)\left(\sum_{j=1}^{N}{\left(y_{j}-\overset{‾}{y}\right)}^{2}\right)}}$$

As before, we recompute the metric after permuting the spots P=1000 times to obtain a p-value.

#### Cell Type region identification (CTRI)

We group together contiguous spots containing cells of the same type into regions (Fig 1g). To accomplish this, we first classified spots based on whether or not they contained a particular cell type using the proportions from SpaDecoder, and our neighborhood smoothed binarization scheme. For each cell type i and spot x we smooth the cell type proportion across its 3D neighborhood of size K, filter out low proportion spots by preserving $x_{i}>T_{1}.\;T_{1}$ can either be explicitly defined or can be selected automatically using Otsu's method for image thresholding [61]. The latter finds the threshold that optimally separates the two classes (presence or absence of the cell type) by maximizing the between class variance and minimizing the within class variance of the proportion of the cell type across spots. We recommend trying both since fixed thresholding performs better for less common or cell types which have low proportions everywhere while the otsu method performs better otherwise. Next, we use connected components analysis to group spots into regions [62]. All spots containing the cell type within a $D_{2-nbd}<=T_{2}\times \text{median}\left(D_{2-nbd}\right)$ of other spots with the cell type were joined into a single region using breadth-first-search (BFS). We filter out regions containing 1 spot.

#### Cell subtype identification in regions (CSI)

We transfer cell subtype annotations from the reference scRNA-seq to the spatial spots (Fig. 1h). In SpaDecoder, we learn a scRNA-seq cell by cell type weight matrix Σ alongwith a cell type proportion matrix V for each spot. We use ΣV for each spot, to derive a scRNA-seq cell by spatial spot mapping M for the entire dataset. We use this to map reference subtype annotations onto the spatial spots with $M^{T}R^{\prime }$ where $R^{\prime }$ is the reference cell subtype annotation we wish to transfer. For each cell type region previously identified from CTRI, we assign a subtype label by comparing the proportions of each of the subtypes in a given spot under the assumption that the subtypes are not colocated in the same spot.

#### Predicting expression of Unseen Genes

In situations when all the genes are not captured spatially, we can use the cell to spot mapping M to predict the spatial expression patterns of all genes profiled by scRNA-seq (Fig 1i). Given the normalized reference scRNA-seq cell by gene expression matrix $B^{\prime }$, we compute $Z=M^{T}B^{\prime }$ where Z is the spot by gene matrix Z containing all genes measured by scRNA-seq. These predicted expression profiles are used for downstream tasks such as finding spatial patterns of relevant markers.

#### Identifying regions with varying cell type proportions across slices

Often, identifying regions of cell type proportion changes across tissue slices is crucial to localizing developmental, disease-associated or tissue configuration induced spatial changes. To this end, we divided the spatial slice into regions and computed the Jenson-Shannon distance (JS) between the discrete probability distributions containing the cell type proportions for the same region across neighboring slices. We then permute the cell type proportions (determined by SpaDecoder) between the spots within each slice and recompute the JS for each permutation to obtain a p-value.

#### Spatio-temporal location prediction

SpaDecoder can be applied to any stack of slices that can be well aligned. This further allows us to map reference scRNA-seq cells spatio-temporally (Fig. 1h) by mapping each scRNA-seq cell to the slice and spot with the highest mapping weight from the mapping matrix M.

### Data

To avoid tissue mismatch between reference scRNA-seq and query spatial datasets, we obtain matching scRNA-seq atlases, spatial datasets along with cell type annotations from published sources. As discussed earlier, lack of ground truth cell type annotations in spots is a major limitation for evaluation of deconvolution algorithms.

While ST simulation algorithms exist, spatial spots have varying number of cells and capturing the distance between spots, the mixture of cell types in each spot, the cell type density across the tissue, and spatial gene expression pattern of real ST tissues is challenging [63]. One solution is to simulate spots from real single-cell resolution ST assays by aggregating the expression profiles of cells in regions of varying sizes. We can then utilize the original published single cell resolution annotations to obtain spot level cell type proportions. A summary of the datasets used is in Supplementary Table 1. Preprocessing is performed in scanpy [56].

#### MERFISH of Mouse Hypothalamus Preoptic Region (Moffitt2018)

Spatial data was obtained from squidpy [60] via sq.datasets.merfish(). Ambiguous cell types and those missing from scRNA-seq data were excluded. For nomenclature consistency with scRNA-seq during evaluation, subtypes of endothelial (1, 2 and 3), immature oligodendrocyte (1, 2), and mature oligdendrocyte (3, 4) major cell types were grouped together into the corresponding major cell type. Blank genes were excluded.

scRNA-seq 10X reference data was downloaded from GSE113576 and associated metadata from Moffitt *et al* Table S1 [37]. Cells having total counts < 2000 or > 25, 000, number of genes < 700 or mitochondrial counts > 10% were excluded. Doublets were separately detected for each sample with scrublet [64] and excluded. Cell types not present in any of the ST samples were excluded.

For visualization of the scRNA-seq UMAP embedding (Supplementary Fig. 5a), cells were normalized with sc.pp.normalize_total, log1p transformed and batch corrected with combat using sc.pp.combat with each sample treated as a separate batch. Cell cycle phase scores and predicted phase for each cell were computed using scanpy.tl.score_genes_cell_cycle with lists of genes in S and G2M phases from (Table S2, Macosko *et al* [65]). Highly variable genes were obtained after excluding mitochondrial, ribosomal protein (Rpl and Rps), and mitochondrial ribosomal protein (Mrpl and Mrps) genes, using scanpy.pp.highly_variable_genes with default parameters. Principal component analysis (PCA) was performed with sc.tl.pca *svd_solver = arpack* and 40 PCs. The 10 nearest neighbor graph was used to perform partition-based graph abstraction (PAGA) which was used for initialization to obtain the UMAP embedding.

#### MERFISH of Mouse Retina (Choi2023)

The V45_integrated.h5ad file was downloaded from Zenodo [66] and separated into individual samples according to region and batch. 25 samples were selected as described in Supplementary Table 1. For the scRNA-seq reference, raw counts were obtained from GSE243413 [42]. Similar to Moffitt2018, cells having total counts < 2000 or > 25, 000, number of genes < 700 or mitochondrial counts > 10% were excluded. We subset to reference cells with “GSE243413 (2023) WT_CD73” condition and visualized the remaining cells on the published embedding. The “majorclass” field was used for cell type annotation.

#### Xenium of Mouse model of Leptomeningeal metastasis (LM) melanoma (Haviv2025)

Raw scRNA-seq reference and spatial data were downloaded from Zenodo [67] with provided umap embedding and the “cell_type” annotation field.

#### Xenium of human breast cancer

The Xenium output bundle was downloaded from 10X [40]. zarr files for the 2 spatial samples were read with read_zarr function from thespatialdata [68] python package. The scRNA-seq reference h5 file alongwith associated annotations and the tSNE embedding for visualization. The ’celltype’ annotation field was used for both scRNA-seq and spatial data. Ambiguous cell types ’Stromal_&_T_Cell_Hybrid’,’T_Cell_&_Tumor_Hybrid’ and those specific to a single modality were excluded. We merged fine grained cell type annotations and ran SpaDecoder and other baselines on course annotations, leveraging fine grained scRNA-seq annotations to highlight our scRNA-spatial mapping and subtype annotation capabilities.

#### Visium of human fetal thymic development

The spatial thymus atlas was downloaded from cellxgene [41] and 16, 17, 18, 19 PCW samples were retained. scRNA-seq reference thymus atlas was also downloaded from cellxgene [69] and 15, 17, and 18 PCW samples were retained. The authors annotate cell types at various resolutions. We use “cell_type_level_0” annotations, excluding exploratory cell types annotated as “see_lv4_explore” but since we wish to study TEC compartments in greater detail, we choose “cell_type_level_2” annotations for Epithelial cells.

### Simulations

Spatial transcriptomic data inherently lacks ground truth cell types in each spot. To this end, we simulate spots from single cell spatial transcriptomic data for some experiments (Figs 2–4 and associated supplementary files). For Fig. 2, we additionally simulate multiple slices from each single spatial sample to recapitulate the profiling of 3D tissue for evaluation purposes. The resulting raw spatial data was input to the preprocessing stage of SpaDecoder.

#### Spot simulation

We normalize the spatial coordinates to the range [0, 1]. Given our spot size parameter $N_{\text{spot}}$, we divide each slice i from left right and top to bottom into square spots of side $\text{stepsize}=\sqrt{N_{\text{spot}}/N_{i}}$ where $N_{i}$ is the number of spots in slice i. Squares to the right and bottom are truncated to the spot size. Intuitively, $N_{\text{spot}}$ is the expected number of cells in the area covered by a spot, assuming uniform distribution of cells throughout the tissue sample, so the square root gives us the side length. Since cells are not distributed uniformly we obtain spots containing different numbers of cells which recapitulates spatial sequencing technologies. Raw spot expression and spot coordinates are defined as the summed raw expression and averaged coordinates respectively of all cells in the spot. Ground truth cell type proportions are the average number of cells of each type in the spot. Default value is N_spot=50. Experiments with other values are indicated.

#### Slice simulation

The first slice in the stack is chosen as the spatial sample. Each subsequent slice i is simulated by perturbing the previous slice i-1. For each spot j, we identify the k+1 nearest neighboring spots including the spot j itself. We randomly select $N_{\text{swap}}$ cells in this neighborhood and randomly shuffle their indices such that their spot assignments and spatial locations are shuffled. The spot expression and cell type proportions are updated accordingly. Default number of neighbors k=10 and simulatedslices=10. We performed experiments with varying $N_{\text{swap}}$ values as described.

### Baseline Methods

#### CARD

CARD [9] was run in R 4.5.0 according to the tutorial. In particular, the steps included createCARDObject with minCountGene=1, minCountSpot=1 and no batch for consistent evaluation and CARD_deconvolution. The cell type proportions from Proportion_CARD were used for evaluation.

#### Cell2location

Cell2location [11] was run according to the tutorial with default parameters. scRNA-seq reference preprocessing involved setting up the Regression Model with cell2location.models.RegressionModel.setup_anndata, RegressionModel, train, export_posterior and obtaining the means_per_cluster_mu_fg. To run cell2location, the steps included cell2location.models.Cell2location.setup_anndata, cell2location.models.Cell2location, train() and export_posterior, with default parameters. The batch size was set to the total number of reference cells. “means_cell_abundance_w_sf” was extracted, normalized and used for evaluation.

#### Tangramsc and Tangram

Tangram and Tangramsc [17] were run according to the provided tutorial. Normalized spatial and scRNA-seq data was input to tg.pp_adatas. tg.map_cells_to_space was run on the GPU with the corresponding cluster key and mode='cells' and 'clusters' for tangramsc and tangram respectively. tg.project_cell_annotations was run with the corresponding cluster key. Cell type predictions in ’tangram_ct_pred’ were normalized and used for evaluation.

### Quantitative Evaluation

Quantitative evaluation was performed by comparing estimated cell type proportions from each of the methods with the cell types as annotated in the original publications with three metrics [20]: average root mean square error (RMSE), Jenson-Shannon divergence (JSD) and Pearson correlation.

#### Root Mean Square Error (RMSE)

For spot j spot in the ith slice, we compute Average $RMSE_{i}=\frac{1}{N_{i}}\Sigma_{j=1\ldots N_{i}}\sqrt{\frac{1}{K}\Sigma_{k=1\ldots K}{\left(GT_{i_{j}}-V_{i_{j}}\right)}^{2}}$ where GT corresponds to ground truth normalized cell type vector and V are those from the deconvolution method. K is the number of cell types, $N_{i}$ the number of spots in the ith slice. Average RMSE is obtained by averaging per spot RMSE across all spots in the slice.

#### Jenson-Shannon divergence (JSD)

Let $D_{p,q}=\sum_{k}\;\left(p_{k}*\text{log}\left(p_{k}/q_{k}\right)\right)$ be the relative entropy between p and q where $p_{k}$ and $q_{k}$ are the respective probabilities. Let $M_{i_{j}}=0.5\times \left({GT}_{i_{j}}+V_{i_{j}}\right)$ be the mean distribution between $GT_{i_{j}}$ representing ground truth normalized proportion of cell types for spot j in slice i and $V_{i_{j}}$ be the corresponding distribution from the deconvolution method. Then, for slice i with $N_{i}$ spots, average $JSD=\frac{1}{N_{i}}\Sigma_{j=1\ldots N_{i}}\left(0.5\times D_{{GT}_{i_{j}},M_{i_{j}}}+0.5\times D_{V_{i_{j}},M_{i_{j}}}\right)$.

#### Pearson correlation

Pandas corrwith with method=’pearson’ was used to obtain Pearson correlation for each spot and averaged across all spots in each slice.

## Supplementary Material

Supplementary Files

This is a list of supplementary files associated with this preprint. Click to download.

- supp.pdf

**Supplementary information.** Supplementary Figures and Tables are attached with this manuscript.

## Data Availability

Publicly available data was used for this study. For Moffitt2018, spatial data was obtained from squidpy [60] via sets.merfish() and scRNA-seq 10X reference data was downloaded from GSE113576 with associated metadata from Moffitt *et al* Table S1 [37]. For Choi2023, spatial data was downloaded from Zenodo [66] and scRNA-seq from GSE243413 [42]. For Haviv2025, raw scRNA-seq reference and spatial data were downloaded from Zenodo [67]. For the human breast cancer dataset [40], the Xenium output bundle was downloaded from 10X. For the human thymic development dataset, the spatial thymus atlas was downloaded from cellxgene [41] and scRNA-seq reference from cellxgene [69].

## Code Availability:

The code for the SpaDecoder package and applications is available at https://github.com/ZhangLabGT/spadecoder.
